# SIDEKICK: Genomic data driven analysis and decision-making framework

**DOI:** 10.1186/1471-2105-11-611

**Published:** 2010-12-30

**Authors:** Mark S Doderer, Kihoon Yoon, Kay A Robbins

**Affiliations:** 1 Department of Computer Science, The University of Texas at San Antonio, San Antonio, TX 78249, USA; 2 Greehey Children's Cancer Research Institute, The University of Texas Health Science Center at San Antonio, San Antonio, TX 78229, USA; 3 Department of Epidemiology and Biostatistics, The University of Texas Health Science Center at San Antonio, San Antonio, TX 78229, USA

## Abstract

**Background:**

Scientists striving to unlock mysteries within complex biological systems face myriad barriers in effectively integrating available information to enhance their understanding. While experimental techniques and available data sources are rapidly evolving, useful information is dispersed across a variety of sources, and sources of the same information often do not use the same format or nomenclature. To harness these expanding resources, scientists need tools that bridge nomenclature differences and allow them to integrate, organize, and evaluate the quality of information without extensive computation.

**Results:**

Sidekick, a genomic data driven analysis and decision making framework, is a web-based tool that provides a user-friendly intuitive solution to the problem of information inaccessibility. Sidekick enables scientists without training in computation and data management to pursue answers to research questions like "What are the mechanisms for disease X" or "Does the set of genes associated with disease X also influence other diseases." Sidekick enables the process of combining heterogeneous data, finding and maintaining the most up-to-date data, evaluating data sources, quantifying confidence in results based on evidence, and managing the multi-step research tasks needed to answer these questions. We demonstrate Sidekick's effectiveness by showing how to accomplish a complex published analysis in a fraction of the original time with no computational effort using Sidekick.

**Conclusions:**

Sidekick is an easy-to-use web-based tool that organizes and facilitates complex genomic research, allowing scientists to explore genomic relationships and formulate hypotheses without computational effort. Possible analysis steps include gene list discovery, gene-pair list discovery, various enrichments for both types of lists, and convenient list manipulation. Further, Sidekick's ability to characterize pairs of genes offers new ways to approach genomic analysis that traditional single gene lists do not, particularly in areas such as interaction discovery.

## Background

**Figure 1:**
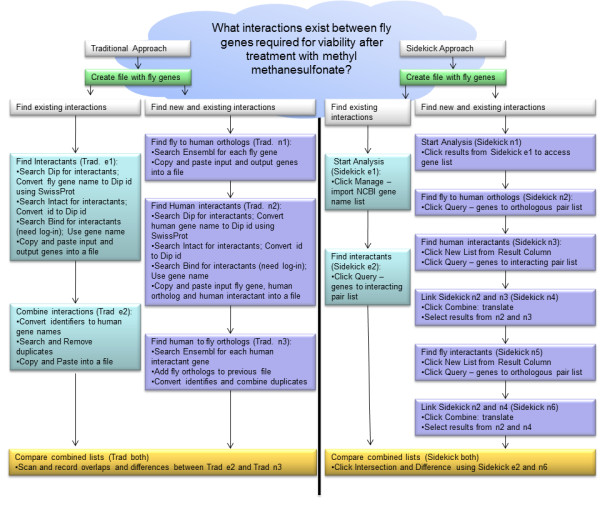
**Comparison of analysis steps performed traditionally and with Sidekick**.

Increasingly, the search for mechanisms and biological processes in complex diseases begins with exploration of data from many sources to incorporate clinical, molecular, and high-throughput genomic data. A scientist might search literature and other databases for candidate interactions, pathways, etc. to hone in on likely candidates for study in the wet lab. The discovery process requires downloading data from several data sources, matching identifiers between data lists, and manipulating lists to match elements from one list with elements from other lists. The left flow chart in Figure 1 shows an example of the traditional process, which is tedious and error-prone when done by hand and which generally requires considerable computational skill to automate.

The right flow chart in Figure 1 summarizes the corresponding process in Sidekick, a web-based genomic decision framework that bridges the work of laboratory and computational scientists. Sidekick enables intuitive query and combination of complex data sources to facilitate research and discovery with no requirement for computational expertise. The framework capitalizes on web services provided by quality data sources and incorporates the researcher's beliefs to weight results. Sidekick handles gene-pair lists as well as gene lists, facilitating the representation of interactions. Interacting pairs involved in important cellular functions are expected to evolve in a coordinated manner in order to preserve these functions [1], thus co-evolution information often provides better insight into physical interactions than simple amino acid sequence analyses. Sidekick enables protein interaction mapping among various species in a simple manner to promote the inclusion of co-evolution information. This paper describes the Sidekick framework and demonstrates its capabilities and flexibility using the problem posed in Figure 1 for illustration.

Much of the previous work in biological knowledge discovery has focused on large-scale top-down discovery rather than bottom-up development of hypotheses. For example, Castellano *et al*. [2] mine information from 5,000 scientific documents using parallel processing in a grid computing environment. Their sample application extracted symptoms and pathologies from the unstructured documents, highlighting the computation power of a grid approach for large-scale discovery using text mining. Pounds *et al*. [3] have developed a tool that determines the statistical significance within groups of gene expression datasets by identifying patterns of association with more than one endpoint analysis. G-SESAME [4] determines gene similarity based on GO terms, while ClueGO [5] and PIPE [6] facilitate mass spectrometry analysis and gene annotation exploration.

An obvious step is to combine multiple focused research tasks into a single tool for knowledge discovery. The DiscoveryNet system [7] facilitates this combination using grid computing for computationally expensive analyses. The system allows scientists to build reusable workflows that can be deployed for use outside the original creator's lab. DiscoveryNet has become an important portion of the InforSense consulting company, which specializes in high-throughput discovery workflows. In one example, Celera used an InforSense workflow to browse, analyze, and integrate clinical data including enzyme linked immunoassay and single nucleotide polymorphism data. While DiscoveryNet is a viable solution for large companies, the software appears to be financially out-of-reach for smaller labs and appears to limit the knowledge exploration to pre-determined workflows rather than allowing the incremental discovery of information to drive the discovery process.

Gaggle [8] in conjunction with Firegoose [9] provides a free plug-in for the Mozilla Firefox web browser that facilitates transfer of information between various bioinformatics websites including KEGG, EMBL, STRING, and DAVID. Users can transfer information to and from local desktop applications such as PIPE and ClueGo that perform specific bioinformatics analyses. Although this approach offers the flexibility of no set workflow order and free availability, the tools require multiple installations and provide no visual representation of the steps required for a particular analysis. Users must parse the output from each web site source. Also, the system does not provide a mechanism for assessing and organizing the user's belief or confidence in the results.

GenePattern [10], like Gaggle and Firegoose, runs within a web browser but also can be downloaded and run locally. Originally created for gene expression analysis, GenePattern also enables single nucleotide polymorphism and proteomics analysis. GenePattern provides fixed workflows that encapsulate analyses processes and allows the development of user-created workflows. GenePattern processes are oriented towards capturing detailed and possibly large-scale computational analysis rather than initial exploration, knowledge discovery, and evaluation of data.

QuExT [11], which focuses primarily on literature searches, retrieves relevant articles given an input set of genes and modifies the order of article relevance to reflect user belief. It initially gives each synonym and gene name equal weight; however, the user can indicate preference for article types by increasing the weight of that synonym or gene name concept, thus changing the results to match the user's belief.

## Implementation

### Overall organization and purpose

Sidekick is a biological knowledge discovery application that focuses on bottom-up discovery and organization of belief. Sidekick combines multiple sources of data for many common research tasks including determination of genes involved in a disease, diseases associated with a gene, gene expression enrichment, Gene Ontology enrichment, chromosome locality enrichment, and interactions. By using web services, Sidekick keeps its information as current as the data sources themselves. The user can save and combine analysis steps to easily document and reproduce results or back track to previous states when investigations in one direction do not produce meaningful results.

Currently Sidekick supports three queries, four filters, several ways to combine results, and methods for saving and restoring workflows and data. Sidekick's modular design using Adobe Flex and Action Script 3 allows programmers to incorporate additional queries, filters, or data sources. Sidekick runs in any browser with the latest Adobe Flash player plug-in. The Sidekick website, http://visual.cs.utsa.edu/sidekick/home.html provides a user's guide and Flash tutorial describing Sidekick's use.

Sidekick has a unique system for managing user belief that makes the user an active participant in assigning confidence measurements to biological discoveries. Users can combine various quality measures provided by different sources to evaluate quality of the analysis. Furthermore, users can incorporate their own biases related to the specific sources of information and particular types and magnitudes of measurements enabled by Sidekick's visualization module and underlying Dempster Schafer [12] confidence source combination.

**Figure 2:**
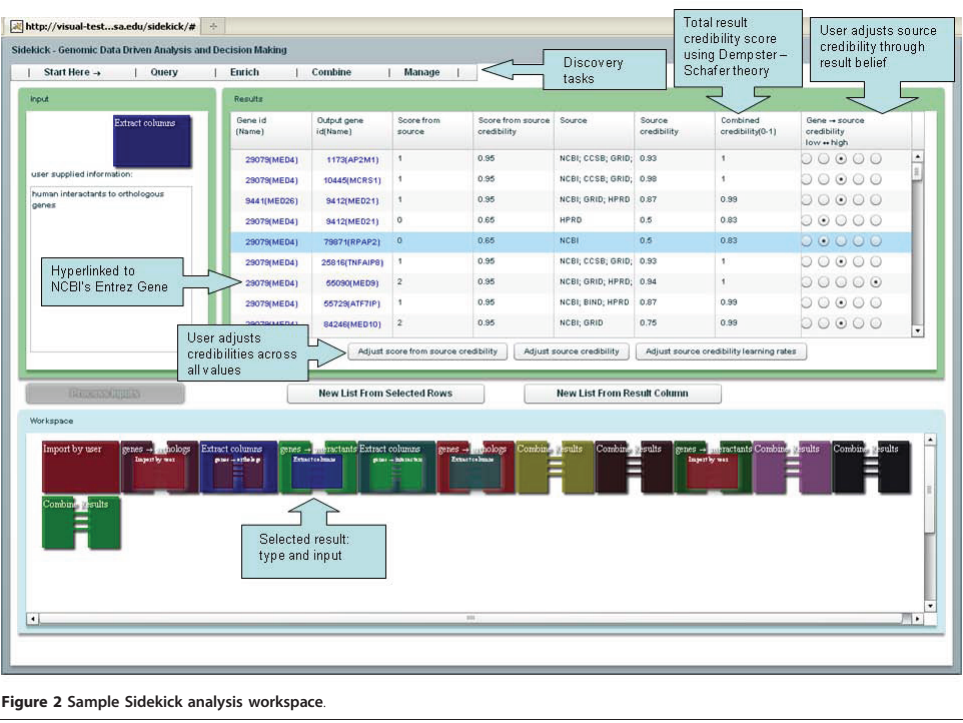
**Sample Sidekick analysis workspace**.

Although genes have many synonyms, Sidekick uses NCBI's gene ID as the common denominator for identifying genes. Currently Sidekick incorporates data from six species (human, mouse, fly, worm, yeast, and zebrafish), with plans to include more. Sidekick is organized around four types of actions: query, enrich, combine, and manage. The query and enrich modules generate a provenance date of download to enable users to identify research results with data source version. The remainder of this section outlines the analysis, sources of information, expected results, and types of confidence measurements that the user can influence based on background knowledge. See Figure 2 for reference during the description.

### Query

*Query: disease/term → gene list *generates a list of genes given a search term by combining results from both NCBI National Center for Biotechnology Information http://www.ncbi.nlm.nih.gov/ and NCIBI the National Center for Integrative Biomedical Informatics http://www.ncibi.org. NCIBI's Gene2MeSH uses a statistical approach to annotate genes reliably and automatically with the concepts defined in MeSH, the National Library of Medicine's controlled vocabulary for biology and medicine. The Gene2MeSH web service returns gene symbols given a MeSH term or MeSH terms for a given gene. Sidekick's *disease/term *→ *genes list *queries Gene2MeSH for the input term and builds the resulting gene list. Each result entry includes a *Score-from-source *representing the significance of the association between the input disease and genes derived from PubMed abstracts. Gene2MeSH returns a p-value for this score.

NCBI's *ESummary *web service produces a gene list given a term (not necessarily a disease term), but does not assign a p-value to the gene-term pair. Sidekick uses a value of 0.05 as a default p-value for NCBI's *Score-from-source*. Since NCBI does not limit search terms to diseases, NCBI's results can be broader than NCIBI's more focused results. The user can choose between *disease only *or *general *within the *NCBI search *input filter to focus on only disease terms or to allow for generalized searches. Sidekick uses NCIBI's p-value when both sources return a particular gene-term pair.

*Query: genes → interacting pair list *searches several data sources for genes believed to interact with the input gene list. The web services that provide the interaction data are NCBI and NCIBI's MiMI [13]. MiMI was created by compiling several publicly available data sources including HPRD [14], IntAct [15], BIND [16], BioGRID [17], MINT [18], CCSB [19], DIP [20], Reactome [21], and MDC [22]. The *Score-from-source *is the number of articles that describe a specific interaction provided by MiMI's web service. The input genes are in the first column and the output genes are the interactants of the corresponding input gene.

*Query: genes → orthologous pair list*, finds orthologs of genes between species from a user specified list. Sidekick uses Ensembl's orthologous gene lists and displays the percent identity between the two orthologs as the *Score-from-source*. The output genes are orthologs corresponding to the input gene.

Sidekick also displays the sources of information for all results. If multiple web services returned the same result, Sidekick lists each source. Using the visualization module and the individual result evaluation tool, users can modify the *Source credibility *and *Score-from-source credibility *based on user confidence in the source and confidence in the score provided by the source. User assigned credibility falls in the range (1, 1), where 1 indicates perfect belief or confidence, 0 indicates neutral belief or confidence, and 1 indicates perfect disbelief The default credibility is 0.5. Sidekick then combines confidence scores into a single *Combined credibility *value, as described later.

Users can sort the query results by columns, and the gene IDs are hyperlinked to NCBI's Entrez website.

### Enrich

Enrichment looks for a common feature among elements of a study set that occurs more frequently than at random as determined by a population set. The difference between random occurrence and the specific occurrence is often encapsulated by a p-value, which can be used as confidence measurement. Sidekick's enrichment modules discover concepts that are enriched in a list of genes or in a list of gene pairs. Sidekick currently supports enrichment by GO terms, mesh terms, gene expression, and chromosomal proximity. The user can sort the results in various ways, and the result genes are linked to NCBI.

*Enrich: genes for GO term *explores the relationships among genes in a given gene list according to their Gene Ontology (GO) annotations. A traditional approach to GO term enrichment takes each GO term and determines if that term is over-represented within the gene study set, as compared to a larger gene set population. The terms in the Gene Ontology are not independent, but rather form a directed acyclic graph with more specific terms as the children of more general parents. For example, mismatch repair is a child of DNA repair. Simple term-for-term analysis does not take into account the potential relationships among different GO terms such as a parent-child relationship. Grossmann *et al*. [23] present a novel approach for detecting overrepresentation of GO terms using parent-child analysis. Their method addresses not only the hierarchical nature of GO terms but also occurrences of the same term in multiple branches of the graph. The less rigorous *Parent-Child-Union *strategy defines the set of parents of a term *t *as the union of genes annotated with *any *parent of *t*. The *Parent-Child-Intersection *strategy reduces the number of enriched terms by defining the set of parents of a term *t *as the intersection of genes annotated with term *t*, counting the genes annotated for *all *of the parents. Grossmann *et al*. conclude the parent child approach avoids many of the false positives that the *Term-For-Term *approach produces.

IEA, Inferred from Electronic Annotation, consists only of evidence from computational analysis and is considered by some as less trustworthy. The *Evidence included *allows for either *Curated Only (no IEA) *or *All Types (include IEA)*. Selecting *All Types *increases the evidence, but perhaps decreases the perceived quality. The *Maximum term hits *allows for either targeted or more general searches.

Each result from enrichment analysis includes the enrichment term, the gene or gene pair found to be enriched for that term, and scores that represent the confidence in the enrichment result. The *Score-from-source *is the p-value representing the likelihood that a subset with shared GO terms happened randomly as compared to the general population. This value and the user's background knowledge form the *Score-from-source credibility *value. Another important factor in enrichment studies is the size of the enriched set relative to the set as a whole. The *Size-of-group *value is the number of genes enriched for the same term. The *Size-of-group credibility *score allows the user to define the importance of this measurement. Sidekick combines all of the credibility scores using Dempster Schafer theory.

*Enrich: for disease terms *uses NCIBI's Gene2MeSH web service to find the disease mesh terms enriched in subsets of the gene inputs. Sidekick only uses the disease category mesh terms from the NCBI's National Library of Medicine. Like GO terms, disease mesh terms are hierarchical. Sidekick uses a modified version of the GO term enrichment algorithm of Grossmann *et al*. to combine mesh terms in parent-children relationships. The outputs are similar to those of GO term enrichment, with the *Score-from-source *as the p-value representing the likelihood that a subset with shared mesh terms happened randomly as compared to the general population.

Gene expression refers to the number of transcripts produced from a gene, which is loosely related to the number proteins produced. Sets of genes that are over-expressed or under-expressed under a specific condition as compared to the population as a whole may be related. The European Bioinformatics Institute provides a web service, Gene Expression Atlas within ArrayExpress [24] that contains curated data for gene expressions under different biological conditions across experiments. The conditions include cell type, developmental stage, and disease state among many others. *Enrich: for gene expression *allows input of a gene list and selection of multiple conditions. ArrayExpress does not directly return a p-value but instead returns the number of experiments in which the gene is up/down regulated for that condition. Sidekick forms the *Score-from-source *by subtracting the number of down-regulated experiments from the number of up-regulated experiments. If the final *Score-from-source *is positive, more evidence for up regulation is present. The other scores are similar to those of the other enrichments.

*Enrich: for chromosomal proximity *displays the gene groups that are most enriched for chromosomal proximity as determined by the number of base pairs separating the start positions of genes. The *Score-from-source *is the p-value of the enrichment indicating the probability that the proximity of the subset of genes could have happened at random.

All of Sidekick gene enrichment analyses allow gene pair lists for input. Sidekick pair-list enrichment analysis finds gene pairs where both elements are enriched for a specific term. As an example, *Enrich: for GO terms *can determine whether a subset of gene pairs localize to the same location in the cellular context. The *Score from source *is the p-value for the enriched subset that contains the genes.

### Combine

The *union *operation finds all genes or gene pairs present in any input list, while *intersect *finds genes or gene pairs present in every input list. The *difference *operation asks the user to designate one of the input lists as the superset and removes all items of the other list from the superset. The *union *and *intersect *operations combine any number of gene lists or gene pair lists from the same species, while *difference *allows only two gene or gene pair lists.

The *translate *operation combines two pair lists when the output of the first list is comparable to the input of the second list. For example, suppose that the first list contains orthologs of human to mouse (pair A-B) and the second list contains interactions of genes in mouse (B-C). Translation produces a gene pair list matching human genes to interacting genes in mouse. For the current modules, column order represents input genes and discovered genes through a two-step process. Eventually Sidekick will support using column order for analyses like transcription factor regulation where the first column holds the transcription factor and the second column holds the target of the transcription factor.

### Manage

File operations include saving and loading an entire workflow, saving and loading gene lists or gene pair lists, importing NCBI IDs or NCBI gene names, and exporting results as a comma separated file. For gene pair lists, the subset can be either a row selection or an output column selection. The resulting subset for row selection is a gene pair list, while column selection produces a single gene list comprised of all genes in the selected rows of the output column with duplicates removed. Extracting rows from an enrichment results set produces the genes contained in the selected groups with duplicates removed. Sidekick uses XML as the underlying file format for most files.

### Belief and confidence for controlling exploration

A user's belief in input sources depends on many factors including the user's background knowledge and view of the data sources. In Sidekick, the level of user belief or credibility for any single score is a value between 1 and 1, where 1 indicates complete acceptance of a result. A credibility value of 1 indicates strong skepticism or alternatively complete belief in the result's negation. Sidekick allows negative values for individual scores so that users can specify that a particular result provides evidence against something. By default, Sidekick assigns a credibility of 0.5 to each score; however, the user is free to adjust this credibility. Sidekick then uses Dempster-Shafer to combine credibility scores to obtain an overall belief credibility score. While the user is able to indicate disbelief with a negative credibility score, the *Combined credibility *indicates belief and therefore always ranges from 0 to 1 where 1 indicates perfect combined belief and 0 indicates lack of belief. These credibility scores allow users to focus on results that are likely to be more significant or more reliable and are useful for sorting results by relevance. The user is also free to ignore completely the tracking of belief.

**Figure 3:**
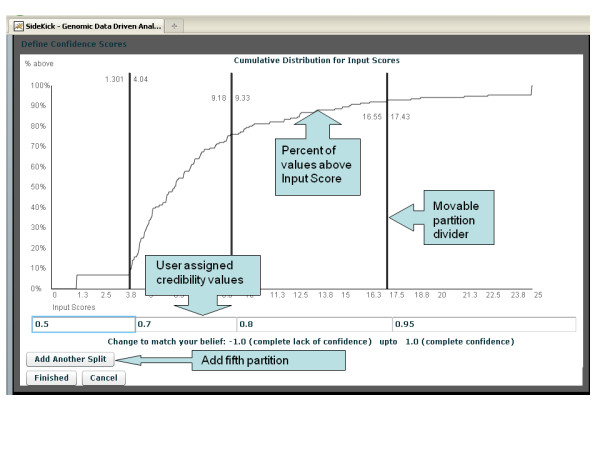
**Sidekick's visualization module for assigning credibilities**.

Each Sidekick operation produces a *Score-from-source *that depends on the type of calculation performed. For example, NCIBI combines many sources in response to mesh-term-to-gene queries and provides a p-value reflecting the weight of evidence for each response. As shown in Figure 3 the visualization module enables the user to adjust credibility scores. The module requires that each score be a non-negative real number, with more significant scores being larger than less significant ones. Sidekick converts *Score-from-source *values into this format, for example, by taking the negative log of a p-value. Sidekick allows the user to control the level of belief in an interactive way. To visualize and assign user belief to a *Score-from-source*, the user presses the *Adjust score-from-source credibility *button. Sidekick displays the normalized scores using a cumulative probability density graph. The same visualization step allows the user to influence the *Combined credibility *by adjusting the *Size-of-group credibility*. The values forming the cumulative probability density graph are the number of elements enriched for each group.

The belief adjustment graph represents both the range of values and the relative density of specific values within the range. Dividers partition the input values and allow users to assign confidence scores to different groups of input values. By increasing or decreasing the number of partitions and moving the partitions, the user can change the confidence score assigned to the input values contained within a partitioned range. Sidekick uses the Expectation Maximization [25] algorithm for determining the initial number of partitions and k-nearest neighbor [26] for clustering to a specified number of partitions (when adding or removing a single partition from the defined number of partitions).

Another way to influence the overall credibility is to evaluate the credibility of the sources that produce a gene or gene pair list (*Source credibility)*. Each source for a given query or filter is initially assigned a weight of 0.5. The user can change this value to reflect belief in the data source in two distinct ways. By pressing the *Adjust source credibility *button, the user can globally change the confidence from 0.5 to reflect increased belief (>0.5) or increased disbelief (<0.5) in the reliability of the specific source.

The user can also influence *Source credibility *by expressing belief or disbelief in individual results from that source. When the user changes the radio buttons at the far right of a result row, Sidekick modifies both the *Combined credibility *for that item and also the *Source credibility *of the site that produced the result. During the analysis, Sidekick iteratively learns its user's beliefs based on the evidence provided by individual credibility decisions. Using a methodology similar to the online training for spam filtering of Goodman *et al*. [27], Sidekick adjusts the *Source credibility score *in a positive or negative direction when the user changes a radio button corresponding to an item from a particular source away from neutral. The learning rates have default settings of (0.030, 0.015, 0.0, 0.015, 0.03), corresponding to adjustment of the five radio buttons to the left or right of neutral. The user can modify these rates by selecting *Adjust source credibility learning rates*.

Sidekick includes credibility measures for all of the filters, queries, and combinations and combines multiple evidence using Dempster Schafer theory (DST) [12] to produce a final *Combined credibility *for each element in a list. DST defines the possible predictive outcomes using the term Universe of Discourse Θ also called the Frame of Discernment. For mutually exclusive outcomes Θ becomes Θ = {T, F} where T = true and F = false. DST also models exceptional situations as Ø. In this case, the Frame of Discernment is Θ = {T, F, Ø}.

Elements of 2^Θ ^(*i.e*., the set of all subsets of Θ) form the class of general propositions in the domain. For our Frame of Discernment the possible sets are {T}, {F}, {TF}, and {Ø}. A function *m*: 2^Θ ^→ (0, 1) is called a *basic probability assignment *if it satisfies:

$$\begin{array}{l} m(Ø)=0,\;\;\sum_{A\subseteq \Theta }m(A)=1\\ \text{and }m\text{(}A\text{)}>=0\text{for all }A \end{array}$$

In other words each possible prediction is assigned a probability, there are no exceptional situations, and collectively the probabilities add up to one. The quantity *m*(A) is defined as A's *basic probability number *and represents our belief in the proposition represented by A. The probability assigned to the set {TF} indicates uncertainty. If there is no uncertainty *i.e. m*(TF) = 0 then *m(*T) + *m*(F) = 1

A belief function assigns to each subset of Θ a measure of our total belief in the proposition represented by the subset. The one-to-one relationship between belief functions and basic probability assignments is given by the following formula:

$$Bel(A)=\sum_{B\subseteq A}m(B)$$

Two pieces of evidence can be combined using DST. Let *m*_1 _and *m*_2 _be basic probability assignments on the Frame of Discernment Θ for two pieces of evidence. Belief is combined by finding the orthogonal sum of *m*_1 _and *m*_2_, *i.e*. *m *= *m*_1 _**⊕ ***m*_2 _and *m*(Ø) = 0

$$\begin{array}{l} m(A)=K\sum_{X\cap Y=A}m_{1}(X)•m_{2}(Y)\\ K^{-1}=\sum_{X\cap Y\neq Ø}m_{1}(X)•m_{2}(Y) \end{array}$$

The quantity log K quantifies the conflict between the two basic probability assignments. A detailed example of combining credibility using Dempster Shafer Theory appears in the supplemental materials file. See Additional file 1 supplemental.pdf

## Results

This section highlights the strengths of Sidekick by reproducing the analysis of InterologueFinder [28], which involves creation of a protein interaction network comprised of known and predicted interactions. As briefly described in Figure 1 InterologueFinder constructed a protein-protein interaction network comprised of known and predicted interactions within five species. Although Sidekick has added a sixth species, we will limit the discussion to the original five species. InterologueFinder inferred interactions in a target species by looking for known interactions in source species and then matching orthologous proteins in the target species. Team biologists then applied coimmunoprecipitation to verify predicted interactions in fly. The biologists' understanding of protein conservation across speciation events enabled confidence in this approach. InterologueFinder also included a quantitative way to compare the interactions. Protein interactions range from weak to strong interactions. InterologueFinder combined several measurements including the experimental support for a known interaction, the species support for known and predicted interactions, and the orthologous support of known and predicted interactions into a single score to represent the believability of both known and predicted interactions. InterologueFinder used a different method of evidence combination than Sidekick does and does not incorporate user belief.

As with Sidekick, InterologueFinder analysis does not focus on maximizing the number of possible interactions by increasing coverage, but rather on generating as high quality interactions as possible. The work was carried out by a team comprised of both computer scientists and biologists, each bringing different perspectives to the analysis task. The computational tasks involved combining a variety of datasets, matching different identifiers for the same proteins, and predicting the protein interactions in one species by analyzing protein interactions among orthologous proteins in other species. The biologists brought specific expert knowledge to the process by determining which data sets should be trusted as high quality and the biological foundation for predicting interactions. They had formulated opinions through an understanding of the process by which the data repository incorporates new information and through a working knowledge of the data used for their own research and its worth as related to their work.

Sidekick enables computational analysis of systems such as InterologueFinder and places researchers in control of their prior biological knowledge and research goals without the need for computational support. InterologueFinder discovered a number of interactions that had not appeared in other data sources (specifically, MED26 MED4, MED26 MED16, and MED26 MED17), which were subsequently verified in the wet lab. Users can easily reproduce the published results of InterologFinder using Sidekick's simple queries and data combination modules in 10 easy steps. We compare the tasks required for both development and use of InterologueFinder and Sidekick. The discovery of multi-species protein networks requires orthologous gene lists and protein-protein interactions for the species to be studied (*e.g*., human, mouse, worm, fly and yeast).

### Typical manual approach (as illustrated by InterologFinder)

Team biologists began the InterologueFinder work with a small list of potential target genes of interest and determined interactions present in publically available data bases (Figure 1 Trad. e1 e2). The computational team members expanded the interaction network by adding interactions present in orthologs based on orthologous relationships between genes specified by Ensembl (Figure 1 Trad. n1 n3). These relationships are identified by Ensembl identifiers that must be mapped to NCBI gene IDs. A computational scientist downloaded, parsed, and remapped these interactions in order to implement InterologueFinder. This process was performed multiple times when Ensembl was updated (Figure 1 Trad. n1 and n3).

InterologueFinder also combined several data sources (BIND, DIP and IntAct). Computational team members downloaded Ensembl synonym tables and converted protein identifiers from the IntAct and DIP databases into Ensembl IDs. The BIND database uses NCBI IDs. The data required remapping to the latest IDs and finding associated protein accession numbers, removing non-protein molecules, filtering for the appropriate species, and removing redundant results. Over the course of developing the application that predicted protein interactions, scientists downloaded, parsed and combined the data sources multiple times because the information became out-of-date (Figure 1 Trad. e1 and n2).

InterologueFinder iteratively processed all five species using the interaction files and the following strategy. For a test gene pair, G1 and G2, that does not have a known interaction in the target species, the procedure was to find orthologs (G1*' *and G2*' *) in another species and check for interactions between the orthologs. InterologueFinder used the orthologous relationship to infer the interaction for G1 and G2 (Figure 1 Trad. n1 n3). Development of the program took many hours of programming and several hours to run. InterologueFinder used this method to identify the predictions MED26 MED4, MED26 MED16, and MED26 MED17 in fly from interactions in human. Scientists selected these gene interactions because of their inclusion in a genome-wide RNAi screen of genes required for viability after treatment with methyl methanesulfonate.

Comparing the lists in InterologueFinder (Figure 1 Trad. both) required a program to compare all known (Figure 1 Trad. e2) and discovered interactions (Figure 1 Trad. n3). When done by hand it is only feasible to check a few interactions for membership in both lists.

### Sidekick's flexible exploratory approach

Sidekick provides a simple *Query: genes *→ *orthologous pair list *that allows users to find the orthologs in a specified species. Behind the scenes, Sidekick uses the same files from Ensembl. However, these files are cached on the Sidekick server. Sidekick only downloads the information when a user requests a new species species combination and refreshes the local copy of the two files weekly to maintain accuracy. Downloading of files is necessary because Ensembl does not offer web services for data retrieval; however the Sidekick server manages the data download (Figure 1 Sidekick n2 and n5)

Sidekick's *Query: genes *→ *interacting pair list *uses NCBI and NCIBI's MiMI's web service to retrieve interaction information. MiMI contains all of the data sources included in InterologueFinder's analysis and several additional interaction data sources. Sidekick doesn't download files, but rather acquires just-in-time information using MiMI web services.

Sidekick's directed research approach enables the user to achieve the same results as a process such as InterologueFinder. For example, using the fly gene MED26 as input into the *Query: genes *→ *orthologous pair list *with *H. sapiens *as the target species, the user finds the fly pair ortholog: 43816(MED26) 9441(MED26) (Figure 1 Sidekick n2). The user can reduce the result to a single gene list by selecting *New List From Result Column*. The single human gene list now forms the input into *Query: genes → interacting pair list*, producing a second gene pair list (Figure 1 Sidekick n3). This pair list contains 45 human interactions. To connect the input column of the orthologs (Figure 1 Sidekick n2) to the output column of the interactants (Figure 1 Sidekick n3), use *Combine*: *translate*: *geneA-geneB translate geneB-geneC → geneA-geneC*. The resulting pair list (Figure 1 Sidekick n4) has 45 fly genes and their orthologous interacting partners in human. After extracting the results column, using *Query: genes *→ *orthologous pair list *(Figure 1 Sidekick n5), and translating between interactants and orthologs, we now have connected the original fly gene with fly interactants (Figure 1 Sidekick n6).

The process finds both known and unknown interactions. To isolate only predicted interactions, simply use the original fly input list and run *Query: genes *→ *interacting pair list *to form a pair list (Figure 1 Sidekick e2). Using (Figure 1 Sidekick n6) as the superset, the difference between interactions found through orthologous transfer and known interactions removes all known interactions leaving only interactions not known in public data bases (Figure 1 Sidekick both). This list not only contains all three of the interactions validated by InterologueFinder, but also 37 other interactions that might warrant further analysis. Figure 2 shows a screenshot of Sidekick at the end of a similar analysis.

### Sidekick's provenance and version tracking

Sidekick maintains dates of data acquisition within each query and enrichment. Sidekick either queries the web source directly or maintains a local copy of the data based on the stated update schedule for each web source; so that the user receives up-to-date data when using Sidekick. Table 1 shows the current data sources and their stated update schedule.

**Table 1:** Update frequency policy for Sidekick's data sources

Sidekick Query/Enrichment	Source	Update Policy
*Query: disease/term → gene list*	NCBI	daily
	NCIBI Gene2Mesh	monthly
*Query: genes → interacting pair list*	NCBI	daily
	NCIBI MiMI	no stated policy
*Query: genes → orthologous pair list*	Ensembl	every 2 months
*Enrich: genes for GO term*	NCBI	daily
	GeneOntology	daily
*Enrich: for disease terms*	NCIBI Gene2Mesh	monthly
*Enrich: for gene expression*	EBI ArrayExpress/Gene	8^th ^of each month
	Express Atlas	
*Enrich: for chromosomal proximity*	NCBI	daily

Provenance information does not give the data source's current version, like Ensembl's current database version of "Ensembl Genes 59". This information is not always available across all data sources. Rather, using the date of acquisition that is associated with a specific data source and maintained across file save and loads, a user is able to maintain information relevant to the age of their analysis as it relates to data source updates.

### Sidekick's extensibility

Sidekick currently supports six genomes, however the framework was constructed with the goal of extending to a variety of genomes. To add an additional species two main areas need to be updated. In the global base class, four lists are included to define different aspects of each species. These include the taxonomy identifier, the formal species name, an informal name for drop-down list display and the group label to use from NCBI's sequence files. When a new species is added to these lists, the front end of Sidekick is modified. The second area to update is the backend file downloading and processing. This includes species specific download and processing of NCBI gene and sequence information, Ensembl orthology information and processing of Gene Ontology information files.

## Discussion

The proliferation of publicly available biological data sources greatly increases potential for discovery but also raises issues of complexity, organization, and reliability for the user. Multiple data sources increase coverage and confidence in the annotated information, but sources are quite varied in their reliability and format, each with strengths and weaknesses. Mathivanan *et al*. [29] evaluated several human protein-protein interaction databases that are available for download to aid researchers in choosing the data for their experiments. The databases included BIND [16], DIP [20], HPRD [14], IntAct [15], MINT [18], MIPS [30], PDZBase [31], and Reactome [21]. They discovered significant variations among these databases in terms of number of genes represented, number of interactions, source of data for the databases, and vocabularies. Sidekick capitalizes on integrated sources such as NCIBI's MiMI, which has compiled an extensive number of databases to increase coverage of many types of information while rectifying vocabulary differences. Many of the data sources covered by Mathivanan *et al*. [29] are present in MiMI and Sidekick.

The Sidekick architecture is extensible, providing an opportunity for integration of new services at the user level. A data source must make its data available through a web service, placing the burden of keeping the data up-to-date on the source itself. The Sidekick web server stages data files that must be downloaded, providing an interface for Sidekick clients. Some analyses are performed on the server, while other analyses are performed on the client, taking advantage of Flex's client-side object-oriented support.

Sidekick manages a variety of credibility measurements to quantitatively describe belief in information and uses Dempster-Shafer's ability to represent uncertainty and lack of knowledge. Credibility, which is more loosely defined than a strict probability, allows users to organize information about importance and to sort by these factors over successive calculations using information from disparate sources. P-values are often used to describe enrichment tasks where probability of the null hypothesis is measured.

Goodman [32] explains the inherent problem of using p-values as a means of measuring belief and uses a Bayesian approach to convert p-values into a probability measurement. However, conversions and combinations of p-values require previous knowledge of the distribution of the input p-values, which may or may not be available.

Dempster-Shafer theory (DST) in combination with Sidekick's visualization of *Score-from-source *addresses the problem of uncertainty, combination of beliefs and conversion of p-values when the underlying distribution is unknown. Dempster-Shafer's theory provides the ability to model and combine certainty even in the presence of uncertainty. Currently, none of the data sources directly conflict with each other. For example, *Query: disease/term → gene list *provides results from both NCBI and NCIBI. The fact that only one source might provide a gene does not mean that the second source has evidence against the inclusion of the gene in the combined list. Rather this situation indicates no evidence for inclusion. However, if two future web sources do conflict, DST manages disbelief as well. When combining conflicting evidence, the strength of the belief and disbelief are combined, with the stronger evidence having a greater effect. Multiple belief functions may be combined by applying DST iteratively for each basic confidence measurement assignment. When multiple web services return the same gene, DST increases confidence in that gene's inclusion in the results list.

The visualization module addresses the problem of p-value conversion without distribution information. For DST to be used, one must have beliefs assigned to each of the values representing the data. Sidekick's visualization and clustering methodology gives the user the ability to manage the conversion from p-value to credibility measures, reflecting prior knowledge in the credibility scores. Once credibility scores are assigned, either as a result of a computation or by the user, Sidekick uses DST for combining these scores into an overall result credibility. A detailed example of combining credibility using Dempster Shafer Theory is provided in the supplemental materials file. See Additional file 1 supplemental.pdf

Provenance and version tracking are incorporated into Sidekick to facilitate repetition of experiments and evaluation of data quality. Given the rate which data is updated, a key aspect to Sidekick's strength is ensuring up-to-date data at the time of downloads. Within Sidekick, provenance information is included in the saved workflows and results.

Sidekick currently supports six genomes, however the framework was constructed with the goal of extending to a variety of other genomes. When an additional genome is added, the developer adds the pertinent information to the species specific lists. Currently this includes the taxonomy id, the formal and informal species names, and the genome sequence group label to use for chromosomal information. If any specific genome is not covered by a particular data source, the lack of information will appear as lack of results in the query or enrichment. This lack of information can be mitigated through the use of *Query: genes → orthologous pair list*. By finding orthologous genes that are covered by existing data sources, the user is able to transfer annotations to the unannotated genome.

## Conclusions

Sidekick offers an easy-to-use web-based, flexible alternative to current research tools. Belief updating through visualization allows scientists to incorporate their own background understanding into the manipulation of data. Gene and gene pair list discovery, enrichment and combination allow accessibility to data and complex biological discovery. Sidekick is unique in its capabilities for scoring and manipulating gene pair lists as well as its user belief management. While the examples of this paper emphasize initial discovery and exploration, Sidekick can also play an important role in interpretation of results obtained from other types of analyses. Often the final stage of an analysis such as clustering produces a gene list. In the discussion of the results, researchers typically apply enrichment analysis to explain how the resultant genes are related. Researchers can further explore these relationships by importing their gene lists into Sidekick and quickly determine interactions, enrichments, and orthology.

We will continue to work with scientists using Sidekick to implement additional modules and services. Not only will these expand analysis involving genes, but also those involving other molecules such as proteins, and protein complexes.

## Availability and requirements

• **Project name: **Sidekick

• **Project home page: **http://visual.cs.utsa.edu/sidekick/

• **Operating system(s): **Platform-independent

• **Programming language: **Flex 3.0 and Action Script 3, Java servlets

• **Other requirements: **Adobe Flash Player 10 and a browser that supports it

• **License: **No license required

• **Any restrictions to use by non-academics: **None

## Authors' contributions

MSD and KAR conceived the framework. MSD developed the framework with input from KY and KAR. All participated in the manuscript development. All authors read and approved the final manuscript.

## Supplementary Material

Additional file 1 **A detailed example of using Dempster Shafer Theory to combine credibility scores**. Click here for file
