# Supplementary material for: SIDEKICK: Genomic data driven analysis and decision-making framework
Source: BMC Bioinformatics. 2010 Dec 30;11:611. doi: 10.1186/1471-2105-11-611 (PMC3022632; doi:10.1186/1471-2105-11-611)
Supplement: Additional file 1 — A detailed example of using Dempster Shafer Theory to combine credibility scores. [file 1471-2105-11-611-S1.PDF]

Supplemental Material

It is helpful to look at an example, where enrichment produces two different subsets of enrichment: one with 5 out of 10 in the general population and the second with the same p-value for 2 out of 10. Table 2 shows possible values of A with two different probability assignments m_1 and m_2.

Table 2 – Basic probability assignments, beliefs and subsets

**A**	m_1	m_2	$Bel_{m_1}(A)$	$Bel_{m_2}(A)$	**subsets used for** $m_1 \oplus m_2$
ϕ	0	0	0	0	{}
{T}	0.5	0.75	0.5	0.75	$\{T_{m_1}\} \cap \{T_{m_2}\}, \{T_{m_1}\} \cap \{TF_{m_2}\}, \{TF_{m_1}\} \cap \{T_{m_2}\}$
{F}	0	0	0	0	$\{F_{m_1}\} \cap \{F_{m_2}\}, \{F_{m_1}\} \cap \{TF_{m_2}\}, \{TF_{m_1}\} \cap \{F_{m_2}\}$
{TF}	0.5	0.25	1.0	1.0	$\{TF_{m_1}\} \cap \{TF_{m_2}\}$

If the p-value was small compared to the other enriched sets the user might assign a belief score of 0.5 to the *Score-from-source credibility* of both sets. However, the same user might believe the set that contains half of the genes from the general population is more believable than the set with only one fifth of the genes. Using the Belief Manager, the user might assign the groups with size of 2 a belief of 0.5 and those of size 5 a belief of 0.75. This translates into Table 3 and 4. To draw connections between Table 2 and Table 3, let m_1 ⇔ *Group size credibility*, m_2 ⇔ *Score-from-Source Credibility*, T⇔ Belief, F ⇔ Disbelief and TF ⇔ Uncertainty. This example shows how the user influences the final combined credibilities based on beliefs about group size in the presence of equal enrichment scores.

Table 3 – DST combination using stronger credibilities

		Group size credibility		
		Belief	Disbelief	Uncertainty
Score from Source Credibility		**0.75**	0	0.25
Belief	**0.5**	0.375	0	0.125
Disbelief	0	0	0	0
Uncertainty	0.5	0.375	0	0.125

K = 1

$m_{score-from-sourceCred} \oplus m_{groupSizeCredibility}$ {Belief} = (1)(0.375+0.125+0.375) = 0.875

$m_{score-from-sourceCred} \oplus m_{groupSizeCredibility}$ {Disbelief}=(1)(0+0+0)=0

$m_{score-from-sourceCred} \oplus m_{groupSizeCredibility}$ {Uncertainty} = (1)(0.125)=0.125

Table 4 – DST combination using weaker credibilities

		Group size credibility		
		Belief	Disbelief	Uncertainty
Score from Source Credibility		**0.5**	0	0.5
Belief	**0.5**	0.25	0	0.25
Disbelief	0	0	0	0
Uncertainty	0.5	0.25	0	0.25

K = 1

$m_{score-from-sourceCred} \oplus m_{groupSizeCredibility}$ {Belief} = (1)(0.25+0.25+0.25) = 0.75

$m_{score-from-sourceCred} \oplus m_{groupSizeCredibility}$ {Disbelief}=(1)(0+0+0)=0

$m_{score-from-sourceCred} \oplus m_{groupSizeCredibility}$ {Uncertainty} = (1)(0.25)=0.25

Sidekick also uses Dempster-Shafer to combine credibility scores to obtain an overall belief credibility score from combined results. While the user is able to indicate disbelief with a negative credibility score, the *Combined credibility* indicates belief and therefore always ranges from 0 to 1 where 1 indicates perfect combined belief and 0 indicates lack of belief. These credibility scores allow users to focus on results that are likely to be more significant or more reliable.

The previous example then becomes an illustration of combining results. Suppose one disease → gene list includes gene A in the results and after the user adjusts the *Score-from-source credibility* and the *Source credibility*, gene A is assigned a *Combined credibility* of 0.5. If the same gene is found in a different disease → gene list and its *Combined credibility* is 0.75, the resulting *Combined credibility* for gene A would be 0.88. If gene B is present in both results with *Combined credibility* of 0.5 for each result, its *Combined credibility* would be 0.75. It is worth noticing, if a third gene C were only present in one result list, its final *Combined credibility* would be the same as the *Combined credibility* in its initial result list. Lack of evidence is not negative evidence in DST.
